# aBVA Procedure by Uniportal Video-Assisted Thoracoscopic Surgery for Right Upper Peripheral Lung Cancer: A Randomized Trial

**DOI:** 10.3389/fonc.2022.828432

**Published:** 2022-02-03

**Authors:** Kaiying Wang, Jian Zhang, Jianglun Li, Langbo Liu, Zhongben Tang, Xiaojun Du

**Affiliations:** Department of Thoracic Surgery, The Affiliated Hospital of Guizhou Medical University, Guiyang, China

**Keywords:** lung cancer, uniportal, VATS, procedure, lobectomy

## Abstract

**Objective:**

This study aims to determine the optimal dividing order of anatomic pulmonary resection under uniportal video-assisted thoracoscopic surgery (uni-VATS) for patients with right upper peripheral lung cancer.

**Methods:**

Patients who met the eligibility criteria were randomly allocated into the aBVA and VAB groups. In the aBVA group, the surgical procedure proceeded from the posterior to the anterior region (from the deeper to the superficial site). In the VAB group, the dissection orders were vein first followed by arterial branches, followed by the bronchus. Clinical data were collected and analyzed.

**Results:**

Sixty patients were randomly allocated to the aBVA group (*n* = 30) and the VAB group (*n* = 30). The operation time in the aBVA group (230.500 ± 68.360 min) was significantly shorter than that in the VAB group (305.600 ± 107.821 min) (*p* = 0.01). The blood loss in the aBVA group (104.000 ± 70.935 ml) was significantly lower than that in the VAB group (391.000 ± 625.175 ml) (*p* = 0.01). Two patients in the VAB group underwent conversion to 2-portal VATS. The number of lymph nodes (13.367 ± 5.436 vs. 10.333 ± 7.279, *p* = 0.072) and lymph node stations (5.067 ± 1.574 vs. 4.467 ± 2.345, *p* = 0.567) were comparable between the two groups. The differences in the postoperative drainage tube time (5.033 ± 3.113 vs. 6.467 ± 4.447 days, *p* = 0.278) and hospital stay (8.233 ± 3.390 vs. 9.433 ± 4.523 days, *p* = 0.361) were not significantly different between the two groups.

**Conclusion:**

Compared with the VBA procedure, aBVA is easier for patients with right upper peripheral lung cancer who undergo uni-VATS lobectomy.

## Introduction

Lung cancer has the second highest incidence and highest mortality rate of cancer in both men and women worldwide (1). It is well known that surgical resection plays an important role in the comprehensive treatment of nonsmall cell lung cancer (NSCLC). In patients with NSCLC who underwent surgery, the right upper lobe had the highest incidence rate (23.8% to 47.0%) among the five lung lobes (2–7). The current National Comprehensive Cancer Network (NCCN) guidelines for NSCLC suggest that for medically operable disease, resection is the preferred local treatment modality (other modalities include stereotactic ablative radiotherapy, thermal ablation such as radiofrequency ablation, and cryotherapy), and that anatomic pulmonary resection is preferred for the majority of patients with NSCLC (8). However, the optimal order of anatomical hilar resections remains controversial. In addition, the NCCN guidelines for NSCLC also suggest that video-assisted thoracoscopic surgery (VATS) or minimally invasive surgery (including robotic-assisted approaches) should be strongly considered for patients with no anatomical or surgical contraindictions (8). With the advantages of direct view, easy learning, reduced operation time and postoperative drainage duration, decreased postoperative pain and hospitalization, diminished inflammatory response, and faster access to chemotherapy (3, 9, 10), uniportal VATS (uni-VATS) has been widely accepted and used. Therefore, in this study, we attempted to distinguish the optimal order of anatomical pulmonary resection under uni-VATS for patients with right upper peripheral lung cancer.

## Materials and Methods

### Study Design

This project was designed as a pilot, prospective, randomized controlled study and was approved by the Human Ethics Committee and the Research Ethics Committee of the Affiliated Hospital of Guizhou Medical University (Guizhou, China; approval no. 2021-475). Written informed consent was obtained from the parents or legal guardians for the use of their data in scientific research at the beginning of enrollment.

### Patient Recruitment

Eligibility criteria included peripheral NSCLC diagnosed by preoperative computed tomography (CT) scan and pathological findings, operable disease confirmed by preoperative evaluation, and male or female patients. The exclusion criteria were as follows: peripheral massive lesion involving the hilar, calcification of hilar lymph nodes, and complications that were planned to be simultaneously managed by surgery or other surgical contraindications that might impact the perioperative outcomes of surgery, such as seriously poor cardiopulmonary function.

### Randomization

Patients who met the eligibility and exclusion criteria were randomly allocated into the aBVA and VAB groups by minimization (11, 12) based on clinicopathological characteristics, including age, sex, pathology, and TNM stage as the eighth edition of the TNM Classification for Lung Cancer (13).

### Surgical Procedure

**Figure 1:**
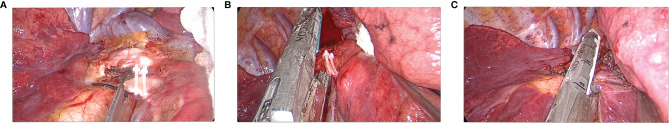
The main steps of the aBVA procedure. **(A)** Cutting the posterior ascending artery (“a” in aBVA), **(B)** stapling the upper bronchus, and **(C)** stapling the upper arterial branches (including variant arterial branches) as well as upper vein simultaneously.

All surgical procedures were performed by the same team. The details of the procedure we used were similar to those described previously (14). However, there were some components that should be reiterated. The incision, approximately 3.0 to 4.0 cm long, was performed at the fifth intercostal space, between the anterior axillary line and posterior axillary line. A small disposable plastic wound protector was used to stretch the incision. A 30°, 10-mm high-definition camera thoracoscope was used to provide a panoramic view and placed at the posterior part of the incision. Wedge resection of the lesions was then performed first in both groups. The main differences between the two groups were the order of the hilar structures to be dissected. In the aBVA group (**Figure 1** and **Video S1**), the procedure proceeded from the posterior to the anterior region (from the deeper to the superficial site). The fissure was stapled first if it was incomplete using the tunnel technique (15), and the posterior ascending artery (“a” in aBVA) was then cut followed by the upper bronchus. The upper arterial branches (including variant arterial branches) were then stapled as well as the upper vein simultaneously with a stapler as the last step.

To shorten the operation duration and reduce the risk of vessel injury, the hilar lymph nodes and surrounding tissue were dissociated from the mediastinum and pushed to the distal end (not removed from the chest right now), which was extracted along with the upper lung in a protective bag when the lobectomy was completed and removed *in vitro* for histopathological examination.

**Figure 2:**
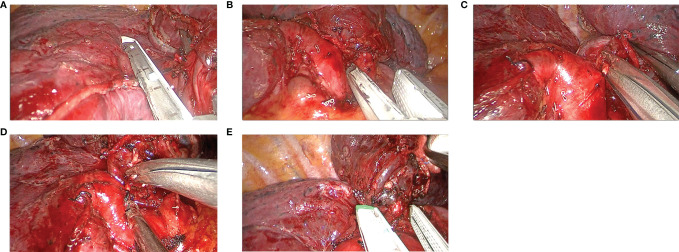
The main steps of the VAB procedure. **(A)** Stapling the upper vein, **(B)** stapling the upper arterial branches, **(C)** cutting the variant arterial branches, **(D)** cutting the posterior ascending artery, and **(E)** stapling the upper bronchus.

In the VAB group (**Figure 2** and **Video S2**), the dissecting orders were as follows: the upper vein was stapled first, followed by the upper arterial branches and variant arterial branches, stapling the fissure if it was incomplete. The posterior ascending artery was then cut, and the upper bronchus was stapled as the last step.

When the fissure was complete, lobectomy was easier and faster in both groups because the artery in the fissure was exposed, and no lung parenchyma was incised. As a rule, in both groups, double ligation was used for all vessels less than 10 mm in diameter; otherwise, a stapler was used. Systemic node dissection was performed to remove the right upper and lower paratracheal, subcarinal, paraesophageal, and pulmonary ligament lymph nodes. At the end of the surgery, one intercostal drain was placed through the incision, as described previously (16), and was removed postoperatively when the daily drainage was <200 ml with no air leakage and sufficient lung expansion on chest X-rays. Patients were usually discharged the day after the chest tube removal and were routinely followed up after 1 week, every 3 months until 2 years postoperatively, and every 6 months thereafter.

### Conversion to Multiportal VATS or Thoracotomy

The surgeons made the decision to convert to multiportal VATS if the operation was difficult to proceed or thoracotomy when uncontrolled bleeding occurred. If conversion to multiportal VATS was required, a 1.2-cm assistant incision at the midaxillary line or another 1.2 cm assistant incision at the posterior axillary line was performed at the seventh intercostal space. When conversion to thoracotomy was needed, anterior and posterior extension of the uniportal incision to about 10 cm in length at the fifth intercostal space was made.

### Data Collection and Statistical Analysis

All clinical data were collected from the institutional database, anesthesia and surgical notes, and medical and nursing records. Descriptive statistics were used to describe the demographic characteristics. Continuous variables were presented as mean ± standard deviation (mean ± SD), and categorical variables are presented as numbers and percentages. When variances were equal, a two-sample unpaired *t*-test with equal variance was used for continuous variables. For unequal variances, the two-sample Wilcoxon rank-sum (Mann-Whitney) test was used. *χ*^2^ or Fisher’s exact test was used for binary categorical data, and results are presented as odds ratios (ORs) and 95% confidence intervals (CIs). Statistical analysis was performed using Stata 15.0 (StataCorp LP). All statistical tests were two sided, and *p* < 0.05 was considered to indicate a statistically significant difference.

## Results

Sixty consecutive patients with right upper peripheral NSCLC were randomly allocated to the aBVA group (*n* = 30) and the VAB group (*n* = 30). The differences in the clinicopathological characteristics between the aBVA and VAB groups were not significant (**Table 1**). The operating time in the aBVA group (230.500 ± 68.360 min) was significantly lower than that in the VAB group (305.600 ± 107.821 min) (*p* = 0.01). Consequently, the blood loss in the aBVA group (104.000 ± 70.935 ml) was significantly lower than that in the VAB group (391.000 ± 625.175 ml) (*p* = 0.01). Two patients in the VAB group underwent conversion to 2-portal VATS because of difficulty in placing the stapler around the superior pulmonary vein due to a lack of angle. The number of lymph nodes (13.367 ± 5.436 vs. 10.333 ± 7.279, *p* = 0.072) and lymph node stations (5.067 ± 1.574 vs. 4.467 ± 2.345, *p* = 0.567) were comparable between the two groups. The differences in the postoperative drainage tube time (5.033 ± 3.113 vs. 6.467 ± 4.447 days, *p* = 0.278) and hospital stay (8.233 ± 3.390 vs. 9.433 ± 4.523 days, *p* = 0.361) were not significant between the two groups (**Table 2**). No uncontrolled bleeding or perioperative death occurred, and no conversion to thoracotomy was needed in either group.

**Table 1:** Differences in clinicopathological characteristics between the VAB and aBVA groups.

Characteristics	VAB group	aBVA group	*p*
Sex			
Female	18	14	0.301
Male	12	16	
Age	59.933 ± 8.103	58.033 ± 7.360	0.345
Pathology			
SCC	4	8	0.197
AC	26	22	
T			
1a	4	5	0.343^*^
1b	12	7	
1c	5	3	
2a	7	14	
2b	1	0	
3	1	1	
*N*			
0	23	24	0.809^*^
1	2	3	
2	5	3	
Stage			
I	16	15	0.356^*^
IIA	4	9	
IIB	5	2	
IIIA	5	4	

SCC, squamous cell carcinoma; AC, adenocarcinoma.

^*^Fisher’s exact test.

**Table 2:** Differences in surgical outcomes between the VAB and aBVA groups.

Characteristics	VAB group	aBVA group	*p*
Surgical time (min)	305.600 ± 107.821	230.500 ± 68.360	0.001^*^
Blood loss (ml)	391.000 ± 625.175	104.000 ± 70.935	<0.001^*^
No. LN removed (*n*)	10.333 ± 7.279	13.367 ± 5.436	0.072
No. LNS removed (*n*)	4.467 ± 2.345	5.067 ± 1.574	0.567^*^
Conversion (*n*)			
No	28	30	0.492^**^
Yes	2	0	
Tube stay (days)	6.467 ± 4.447	5.033 ± 3.113	0.278^*^
Hospital stay (days)	9.433 ± 4.523	8.233 ± 3.390	0.361^*^

LN, lymph nodes; LNS, lymph node station.

^*^Wilcoxon rank-sum (Mann-Whitney) test; ^**^Fisher’s exact test.

## Discussion

The current NCCN guidelines for NSCLC suggest that anatomical pulmonary resection is preferred for the majority of patients with NSCLC (8). However, the optimal order for anatomical resection remains controversial. Traditionally, it has been suggested that the pulmonary vein be cut first to avoid dissemination of tumor cells, which could consequently lead to blood micrometastasis and treatment failure (17–21). However, other studies concluded that the sequence of ligation of pulmonary vessels did not seem to influence oncological outcomes or survival (22–24). Despite the controversy, we still performed wedge resections first for the sake of clarity in the present study.

In addition, the NCCN guidelines for NSCLC suggest that VATS or minimally invasive surgery (including robotic-assisted approaches) should be strongly considered for patients with no anatomical or surgical contraindictions (8). With the advantages of direct view, easy learning, less operation time and postoperative drainage duration, decreased postoperative pain and hospitalization, diminished inflammatory response, and faster access to chemotherapy (3, 9, 10), uni-VATS has been widely accepted and used. Therefore, anatomical right upper pulmonary resection was performed using uni-VATS in this study.

The results of this study demonstrated that the operation time (230.500 ± 68.360 vs. 305.600 ± 107.821 min, *p* = 0.01) and blood loss (104.000 ± 70.935 vs. 391.000 ± 625.175 ml, *p* = 0.01) in the aBVA group were significantly shorter than those in the VAB group. These results were in accordance with those of a previous retrospective study by Zhai et al. (22). These advantages may be attributed to the change in the hilar cutting order. It is well known that in the VAB procedure, the upper pulmonary vein is the most difficult structure to divide first with a stapler through a single incision because it is difficult to achieve better angles for stapler insertion. Many solutions have been attempted, for example, using curved-tip staplers or polymer vascular clips, ligation of the vein using sutures, and cutting the upper arterial branches first (9, 10, 14). When the aBVA procedure is used, it is easy to cut the posterior ascending artery first and the upper bronchus with a stapler through a single incision because they are farther away from the incision. It is easier then to cut the upper pulmonary vein as well as the upper arterial branches (including variant arterial branches), as they have increased degrees of freedom.

The results of this study also showed that the number of lymph nodes (13.367 ± 5.436 vs. 10.333 ± 7.279, *p* = 0.072) and lymph node stations (5.067 ± 1.574 vs. 4.467 ± 2.345, *p* = 0.567) were comparable between the two groups. The differences in the postoperative drainage tube time (5.033 ± 3.113 vs. 6.467 ± 4.447 days, *p* = 0.278) and hospital stay (8.233 ± 3.390 vs. 9.433 ± 4.523 days, *p* = 0.361) were not significantly different between the two groups. This implies that the aBVA procedure can achieve short-term surgical outcomes similar to those of the VAB procedure.

The present study had some limitations. It failed to compare the two procedures in patients with central lung cancer and lacked the results of long-term surgical outcomes. Further investigation is required to address these issues.

## Conclusion

In conclusion, for patients with right upper peripheral lung cancer, compared with the VAB procedure under uni-VATS, the aBVA procedure is easier and can achieve the same short-term surgical outcomes; therefore, it is worth promoting the application of the aBVA procedure in clinics.

## Data Availability Statement

The raw data supporting the conclusions of this article will be made available by the authors, without undue reservation.

## Ethics Statement

The studies involving human participants were reviewed and approved by the ethics board of the Affiliated Hospital of Guizhou Medical University. The patients/participants provided their written informed consent to participate in this study.

## Author Contributions

KW, JZ, LL, JL, ZT, and XD analyzed and interpreted the data. KW, LL, and JL were major contributors in writing the manuscript. KW, ZT, and XD confirm the authenticity of all the raw data. All authors read and approved the final manuscript.

## Funding

This study was partly funded by the Beijing Xisike Clinical Oncology Research Foundation (grant no. Y-Q201801-006) and the Science and Technology Fund Project of the Health Commission of Guizhou Province (grant no. Gzwjkj2020-1-111).

## Conflict of Interest

The authors declare that the research was conducted in the absence of any commercial or financial relationships that could be construed as a potential conflict of interest.

## Publisher’s Note

All claims expressed in this article are solely those of the authors and do not necessarily represent those of their affiliated organizations, or those of the publisher, the editors and the reviewers. Any product that may be evaluated in this article, or claim that may be made by its manufacturer, is not guaranteed or endorsed by the publisher.

## Supplementary Material

Supplementary Video 1 The main steps of the aBVA procedure.

Supplementary Video 2 The main steps of the VAB procedure.
